# En1 and Wnt signaling in midbrain dopaminergic neuronal development

**DOI:** 10.1186/1749-8104-6-23

**Published:** 2011-05-10

**Authors:** Maria TM Alves dos Santos, Marten P Smidt

**Affiliations:** 1 Rudolf Magnus Institute of Neuroscience, Department of Neurosciences and Pharmacology, University Medical Center Utrecht, Universiteitsweg 100, 3584 CG Utrecht, The Netherlands

## Abstract

Dopaminergic neurons of the ventral mesodiencephalon are affected in significant health disorders such as Parkinson's disease, schizophrenia, and addiction. The ultimate goal of current research endeavors is to improve the clinical treatment of such disorders, such as providing a protocol for cell replacement therapy in Parkinson's disease that will successfully promote the specific differentiation of a stem cell into a dopaminergic neuronal phenotype. Decades of research on the developmental mechanisms of the mesodiencephalic dopaminergic (mdDA) system have led to the identification of many signaling pathways and transcription factors critical in its development. The unraveling of these pathways will help fill in the pieces of the puzzle that today dominates neurodevelopment research: how to make and maintain a mdDA neuron. In the present review, we provide an overview of the mdDA system, the processes and signaling molecules involved in its genesis, with a focus on the transcription factor En1 and the canonical Wnt pathway, highlighting recent findings on their relevance - and interplay - in the development and maintenance of the mdDA system.

## The mesodiencephalic dopaminergic system

The mesodiencephalic dopaminergic (mdDA) system has been the focus of intense scientific research due to its involvement in numerous behavioral and neurological disorders and thus its clinical relevance. The neurotransmitter dopamine (DA) is present in different areas of the brain, such as the hypothalamus, the olfactory bulb and the mid-forebrain. In this last area, mdDA neurons are the main source of dopamine in the mammalian central nervous system (CNS), attributable to two ventral groups of neurons: the substantia nigra pars compacta (SNc) and the ventral tegmental area (VTA) [1-3]. The main innervation targets of mdDA neurons are the basal ganglia. The neurons of the SNc innervate the dorsolateral striatum and caudate putamen forming the nigrostriatal pathway. Neurons of the VTA project to the ventral striatum (nucleus accumbens, amygdala and olfactory tubercle) as part of the mesolimbic system and establish additional ascending connections to the prefrontal cortex forming the mesocortical system. These ventral midbrain nuclei modulate specific brain functions according to its distinct projection fields. The SNc is involved in the control of voluntary movement and body posture, and its selective degeneration leads to Parkinson's disease (PD) [4,5]. The mesocortical and mesolimbic systems, on the other hand, are involved in the modulation and control of cognitive and emotional/rewarding behaviors, and their dysfunction is involved in the pathogenesis of various affective disorders, such as addiction [6-8], depression [9] and schizophrenia [10,11]. Drug abuse, depression and PD constitute highly common health disorders, which explains the intense research in recent years on the mechanisms and factors involved in the generation and survival of mammalian mdDA neurons.

### mdDA neurogenesis and differentiation

**Figure 1:**
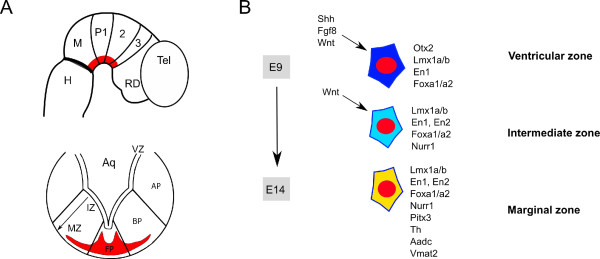
**Spatial and temporal developmental stages leading to mesodiencephalic dopaminergic neurogenesis**. **(A) **Sagittal and coronal schematic sections showing the region in the developing central nervous system where mesodiencephalic dopaminergic (mdDA) neurons are born. Anterior-posterior patterning leads to the genesis of morphogenetic domains: telencephalon (Tel), rostral diencephalon (RD), midbrain (M) and hindbrain (H), whereas dorsal-ventral patterning results in crosswise subdivisions in the brain: floor plate (FP), basal plate (BP), alar plate (AP) and roof plate. The mdDA area encompasses the midbrain and prosomeres (P) 1 to 3. The floor plate is divided in three main areas: the ventricular zone (VZ), the intermediate zone (IZ) and the marginal zone (MZ). **(B) **Molecular cascades leading to mdDA neurogenesis, illustrated by three different stages from top to bottom. The key genes driving mdDA development are represented. En1 and Wnt signaling are required already in early development, being essential throughout mdDA development, from early patterning up to the induction of mdDA neurons. Although we placed En1 in all these developmental stages, a molecular characterization of how En1 contributes to each of these has not yet been performed. It remains to be seen as well whether Wnt signaling is active in a settled mdDA neuron (after embryonic day (E)14). The progenitor pool is located in the VZ and its progeny migrates to the IZ, where it differentiates into post-mitotic mdDA precursors, expressing Nurr1 and L-aromatic amino acid decarboxylase (Aadc). Later on, after E12, mdDA neurons start to differentiate, expressing mdDA key identity genes like *Pitx3, Th *and *Vmat2*. The differentiated settled mdDA neurons localize in the MZ.

The development of an organ, such as the midbrain, implies the sequential occurrence of developmental cascades over time, while these might overlap in time and space [12-14]. During early neuronal induction and patterning, a precise molecular coding along the anterior-posterior and dorsal-ventral axis in the developing neural tube provides positional cues that are crucial in pattern formation [15-17]. Anterior-posterior patterning leads to the genesis of morphogenetic domains - forebrain, midbrain, isthmus and hindbrain - whereas dorsal-ventral patterning results in crosswise subdivisions in the brain throughout the neuroaxis - floor plate, basal plate, alar plate and roof plate [16,18,19] (Figure 1A).

The successful regionalization of the early CNS is necessary for the subsequent correct commitment and positioning of mdDA neurons later on in development [17]. The creation of the isthmus, a neuroepithelial signaling center localized at the midbrain-hindbrain boundary (MHB), together with the ventral signaling center from the floor plate, are essential for pattern formation and the generation of mdDA neurons in the developing embryos [13,15,17]. The isthmus is characterized by the expression of the fibroblast growth factor 8 (Fgf8) and the floor plate by the expression of sonic hedgehog (Shh) [20,21]. The intersection of these secreted factors provides the positional information that determines where the mdDA progenitor domain will arise [15,20,22-24]. While Fgf8 acts as an anterior-posterior morphogen [25], the dorsoventral axis is determined by the ventralizing Shh in the floor plate and the dorsally secreted bone morphogenetic proteins by the roof plate [26-28]. The isthmus is established by the opposing expression domains of two transcriptional repressors: Gbx2 in the presumptive hindbrain and Otx2 in the presumptive mid/forebrain [12,29]. Although Otx2 and Gbx2 are not necessary for the induction of MHB genes (such as *Fgf8*), they are essential for the correct positioning of their expression domains [30]. Other factors as well are involved in the induction and maintenance of mdDA progenitors in the ventral midbrain: the Wnt factors [31], Engrailed (En)1 and En2 [32], Pax2/5 [33,34], Lmx1a, Msx1 and Lmx1b [35,36], and Foxa2 [37,38]. For comprehensive reviews see [39-41]. The interplay of all these factors in the ventral midbrain forms a grid of graded cues in which neural progenitors follow different cell fates depending on their position.

mdDA precursors will eventually become fully differentiated mdDA neurons when they start expressing an array of genes that are essential for DA signaling. These key factors comprise the enzymes tyrosine hydroxylase (Th) and the L-aromatic amino acid decarboxylase (Aadc), which catalyze the conversion of L-tyrosine to L-DOPA and L-DOPA to dopamine, respectively; the vesicular monoamine transporter (Vmat2), which is required for vesicular storage and release of dopamine; and the dopamine transporter (Dat), involved in the re-uptake of DA from the synaptic cleft. These proteins, among others, are essential in the make-up of dopaminergic neurons. Several transcription factors, such as Nurr1, Pitx3, Lmx1b, are critical for the specification of neuronal identity (that is, for the expression of the above mentioned mdDA-specific factors) and maturation and survival of postmitotic mdDA neurons [35,42-44].

Around mouse embryonic day (E)10, proliferative mdDA progenitors present in the ventricular zone (VZ) begin migrating ventrally along radial glia towards the pial surface [45,46] (Figure 1B). These migrating precursors, besides continuing to express a large set of genes from early mdDA progenitor specification, such as those encoding En1/2, Lmx1a/b and Foxa1/2, start expressing Aadc [14] and, at E10.5, the Nurr1 orphan nuclear receptor [42], after which they exit the cell cycle and become postmitotic [47] (Figure 1B). At this stage these cells should be considered postmitotic mdDA precursors since they are not yet fully differentiated mdDA neurons [48]. Th-positive cells were first reported to appear in the mouse ventral midbrain between E9.5 and E11.5 [49,50]. At this time-point the mdDA precursors become dopaminergic neurons as they also acquire the expression of the homeodomain transcription factor Pitx3 [35,45,51]. mdDA neurogenesis peaks around day E12.5 and declines thereafter [50,52]. Once the ventricular-to-pial migration is accomplished by E13, the mdDA neurons take up their position corresponding to the future SNc and VTA [45]. Hereafter, until the first postnatal weeks, the mdDA neurons start extending axonal outgrowths towards their target projection areas within the striatum and cortex [45,53].

To assume that not every cell in the mdDA system is the same, following exactly equal transcriptional programs, is plausible if we consider the existence of a positional grid of signals during neuronal development, each coordinate specifying a different (if modest at times) cell program. This existence of neuronal subsets within the ventral midbrain is now an accepted fact; for example, one difference between the SNc and the VTA is that they present a different temporal order of *Th *and *Pitx3 *gene expression [54]. This difference, together with other differential aspects, might explain why the SNc is more vulnerable than the VTA to neuronal degeneration [4,5,55]. For detailed reviews on mdDA neurogenesis, see [56-58].

## Engrailed

Engrailed proteins are bifunctional homeodomain transcription factors [59], highly conserved throughout the animal kingdom [60,61]. In the murine genome there are two engrailed paralogs, En1 and En2, both essential in embryonic development. En1 is already expressed at the one-somite stage (E8) while En2 expression appears half a day later [62]. The expression domain of engrailed (En1/2) comprises the neuroepithelium of the posterior midbrain and anterior hindbrain, which will later give rise to the cerebellum, the colliculi and the ventral midbrain nuclei [63]. En1 is expressed highly by all mdDA neurons from the moment they start to differentiate (around E11) and continuously into adulthood, whereas En2 is strongly expressed in only a subset of them [32,64]. Outside the CNS, En1 is expressed in the cranial mesenchyme, the mandibular arches, the vagus nerve, the dorsal root ganglia, the sympathetic ganglia, the somites, the heart, the cloaca, and the tail and limb buds [64-67]. In the adult brain, both are expressed in the pons and the substantia nigra and En2 alone is found in cerebellar cells [62,67-69].

**Figure 2:**
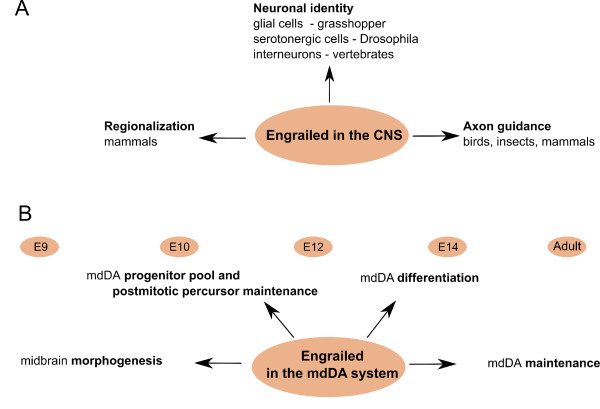
**The impact of the engrailed genes in the development of the central nervous system and the mesodiencephalic dopaminergic system**. **(A) **Engrailed proteins are key players in diverse processes during embryonic development of the central nervous system (CNS), including patterning, axonal guidance and neuron specification. **(B) **Engrailed proteins are essential in mesodiencephalic dopaminergic (mdDA) neuron development from an early stage, where they are involved in morphogenesis and mdDA neurogenesis, and in the adult, where they play a role in mdDA neuron maintenance E, embryonic day.

Today, after two decades of research, a multitude of evidence has been produced establishing engrailed proteins as key players in the embryonic development of the CNS (Figure 2A). Up until now, it has been shown that they are involved in multiple developmental processes: the regionalization in early embryogenesis, including isthmic organization [13,67,70-72]; neuronal identity, such as the control of glial-neuronal fate in the grasshopper [73] and serotonergic midline neurons in *Drosophila *[74]; axonal outgrowth and pathfinding in insects, birds and mammals [75-80]; and the identity specification of subsets of vertebrate interneurons in the spinal cord [68,81]. Engrailed proteins, besides functioning as transcription factors (localized in the nucleus), have also been characterized as secreting survival factors [80,82-84], where about 5% of the engrailed protein is found associated with membrane vesicles, becoming secreted and internalized by cells [82,85,86]. Furthermore, En1 secretion appears to be a regulated process [87]. These studies have shown that engrailed proteins can function as signaling molecules, acting in a paracrine manner in mdDA neurons. In a recent study, mdDA neuronal loss in *En1*^*+/- *^adult mice was antagonized by En2 recombinant protein infusions in the midbrain [88], showing both the ability of engrailed to act as a signaling molecule and suggesting a biochemical equivalence between En1 and En2.

### Engrailed in the development of mdDA neurons

The spatial and temporal expression of En1 in the brain, as mentioned above, is mainly restricted to the mid-hindbrain junction and coincident with the development and maintenance of mdDA neurons. To elucidate a gene's role within a certain biological system, such as the dopaminergic system in the midbrain, the study of its natural mutant (when available) or its engineered knockout is essential. The brain phenotypes of *En1 *and *En2 *mutants are quite different despite their similar expression patterns and structurally related protein products [67]. The *En2 *single mutant is viable and fertile and exhibits a mild cerebellar phenotype involving a reduction in the size of the cerebellum and colliculi with an alteration in its folding pattern, while the mdDA population remains normal [69,89,90]. Generated *En1 *null mutant mice die at birth and have multiple defects, such as abnormal forelimbs and sternum and a deletion of hindbrain tissue resulting in loss of most of the cerebellum and colliculi [67].

The mid-hindbrain phenotype in *En1 *mutant mice, together with corroborative data from other mutants in which En1 expression is absent, such as the *Wnt1 *mutant [91], clearly pinpoint En1 as a critical protein for normal development of mid-hindbrain structures, already starting from its earliest expression period in the neural tube. However, detailed molecular characterization of the mdDA system in the *En1 *single-null mutant is lacking in the literature, the focus having been thus far on the *En1/En2 *double knockout.

A few studies have shown the biochemical equivalence between En1 and En2. En2 can functionally replace En1 when knocked-in to the En1 locus, allowing for normal midbrain development and survival of the murine *En1 *null mutant [92]. This suggests that the two paralogs are maybe redundant and functionally equivalent [60]. Functional equivalence across phyla has also been confirmed by using *Drosophila *engrailed (en) to replace murine En1 [93]. However, neither en nor En2 could rescue the limb defects caused by loss of En1 function [93]. It is generally accepted that the main functional differences between the *engrailed *null mutants are due to their different temporal and spatial expression and not to different molecular properties [51]. However, En1 and En2 differ substantially in their compensatory abilities to maintain ventral midbrain dopaminergic neurons. The complete loss of mdDA neurons only occurs when all four alleles are deleted (*En1*^*-/-*^*; En2*^*-/-*^) [32]. This study by Simon *et al. *study describes that mdDA neurons are induced, become postmitotic and express Th, but this entire mdDA population is lost by E14 due to cell loss [32]. They compare all intermediate genotypes of En1/En2 and show that engrailed is required in mice for the survival of dopaminergic neurons of the SNc and VT in a gene dose-dependent manner, with one *En1 *allele sufficient to produce a wild-type phenotype, while one *En2 *allele cannot [32]. However, in the *En1*^*-/- *^mutant there seems to be no significant alterations in the organization of the mdDA system at birth, with the number of midbrain dopaminergic neurons remaining unaffected [32,67]. Furthermore, the engrailed requirement for the survival of mdDA neurons is cell-autonomous and not caused by the large deficiency of the surrounding midbrain tissue [51].

In more recent studies focusing on the *En1*^*+/- *^genotypes, the *En1*^*+/-*^; *En2 *^*-/- *^mutant showed no apparent embryonic phenotype, while the adult had a phenotype resembling key pathological features of PD [94]. The postnatal mutant mice showed a specific and progressive degeneration of dopaminergic neurons in the substantia nigra during the first 3 months (other DA groups in the mesodiencephalon were not affected), resulting in diminished storage and release of dopamine in the caudate putamen accompanied by motor deficits (akin to akinesia and bradykinesia), and a lower body weight [94]. Interestingly, another study shows that in *En1*^*+/- *^animals, in an *En2 *wild-type background, the number of dopaminergic neurons in the SNc and VTA slowly decrease between 2 and 6 months after birth [88]. This reduction in dopaminergic neurons is stronger in the SNc. The dopaminergic neuronal cell death was paralleled by a substantial decrease in striatal DA, impaired motor skills, and listless behavior. Together, these studies demonstrate that engrailed genes have both important embryonic and adult physiological functions (Figure 2B), and that their deregulation leads to progressive dopaminergic cell death in the adult [88], a characteristic of PD pathology.

### En1 and Parkinson's disease

The pathological hallmark of PD is the selective and progressive degeneration of dopaminergic neurons in the SNc [5]. En1 does affect the survival of this population during embryonic development [88,94]. Furthermore, the deletion of one *En1 *allele leads to massive cell death in the mdDA system of young adult mice [88,94], making *En1 *a potential gene in the molecular cascade leading to progressive cell death in the neuropathology of PD. Recently, Sgado *et al. *[95] observed that *En1 *heterozygous adult mice present a significant reduction in striatal DA levels accompanied by a reduction in the number of nigral dopaminergic neurons, corroborating Sonnier's work [88]. Furthermore, the En1^+/- ^mice show motor deficits together with anhedonia, decreased social interactions and depression-like behaviors, which are reminiscent of symptoms observed in PD [96]. In PD, apoptosis is viewed as the mechanism leading to nigral DA cell death [97-99]. Interestingly, in the absence of both *Engrailed *genes, mdDA neurons undergo caspase-3-mediated apoptosis [51]. Noteworthy, α-synuclein, a protein implicated in PD [100], seems to be regulated by engrailed [51,101]. In a recent study, genetic variants in the *En1 *gene showed a significant association with PD, indicating that these polymorphisms are potential genetic risk factors for sporadic PD [102]. All the evidence so far points to the core importance of En1 in the maintenance of mdDA neurons, making the *En1 *mutant a plausible model for PD research.

## Wnt signaling

A small number of signaling pathways are vital and used repeatedly in the developmental processes of all metazoa. One such core pathway is the Wnt signaling pathway. Wnt signaling was initially characterized in the late 1980s during embryonic development studies of *Drosophila melanogaster*, when it became clear that it was a key event for segmental and spatial organization of the body plan (reviewed in [103]). The term 'Wnt' is derived from a combination of the *Drosophila *wingless (wg) and the mouse homolog Int1 (subsequently Wnt1) proteins [104,105]. Wnts refer to the extracellular ligands of the pathway, which comprise a family of secreted glycolipoproteins able to interact with cell surface receptors eliciting a variety of intracellular responses. Being lipid modified, Wnts are hydrophobic in nature and localize preferentially in the extracellular matrix and cell membranes [106,107].

To date, 19 distinct Wnts have been identified in mammals [108]. Wnt gene diversity is also observed in other animals like *Drosophila, Caenorhabditis elegans *and *Xenopus*, as well as in the ancient metazoan phylum Cnidarians [109], showing not only that Wnts are evolutionarily conserved but that Wnt gene diversity arose early in evolution and is in itself required for successful animal development. Indeed, Wnt signaling is crucial in embryonic development, from gastrulation and early pattern formation to organogenesis [110], and in adult organisms, where it plays a central role in the maintenance of tissue homeostasis and stem cell regulation [111]. Namely, Wnt signaling controls diverse processes, such as cell proliferation, self-renewal, cell polarity, cell death and cell fate specification [112-115]. Wnts can also function as morphogens, acting in both short- and long-range signaling, modulating target cells in a dose- and distance-dependent manner [116-119]. This pivotal and complex role of Wnt signaling in such a myriad of biological processes implies that its deregulation leads to disease, such as cancer, congenital disorders and degenerative diseases [120-124].

The complexity of Wnt signaling is due, in the first place, to the large variety of ligands and receptors involved in Wnt signal transduction, allowing for an impressive number of possible ligand-receptor interactions [125]. For example, there are the frizzled receptors (ten Frizzled (Fz) receptor homologs in mammals) and the recently described non-Fz receptors, the RYK and Ror/Tyr kinases (reviewed in [126,127]). Secondly, Wnt-receptor interactions can produce a variety of intracellular responses, since Wnt target genes are diverse and context-dependent [115,128]. The Wnt signaling pathways have been characterized as either canonical or non-canonical. For extensive and up-to-date reviews on the Wnt receptors and pathways, see [126,129,130]. In this review the focus will be primarily on the canonical pathway.

### Canonical Wnt signaling

The best understood and most extensively studied of the Wnt pathways is the β-catenin-dependent Wnt signal transduction pathway (Wnt/β-catenin), otherwise known as the canonical pathway. This pathway relays its signals by stabilizing β-catenin protein in the nucleus where it will be part of transcriptional complexes mediating key developmental gene expression programs [115]. Besides being the key factor in Wnt canonical signaling, β-catenin functions as a structural adapter protein linking cadherins to the actin cytoskeleton, in particular interacting with both E-cadherin and α-catenin to mediate cell adhesion [131]. The focus of past and recent research on canonical Wnt signaling is explained by the imperative role it has in development and disease, and in stem cell specification and maintenance in various tissues and organs, including the brain (reviewed in [112,132-134]). Wnt-β-catenin signaling inactivation in vertebrates has also been implicated in other diseases, such as heart disease [135] and Alzheimer's [136-139]. The range of action of Wnt/β-catenin signaling spans a few hours, and that is why its activity is observed mainly in contexts of cell-fate determination and tissue homeostasis [140].

### Canonical signaling mechanism

**Figure 3:**
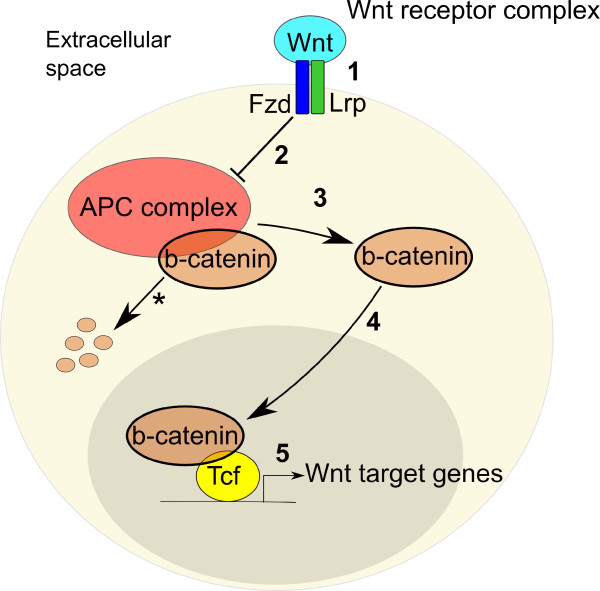
**Canonical Wnt signaling mechanism**. (1) Wnts bind to Frizzled (Fz) transmembrane receptors and low-density lipoprotein receptor-related protein (Lrp) co-receptors (2) triggering the dissolution of the 'β-catenin destruction complex', resulting in (3) β-catenin not being marked for degradation (asterisk) thereby accumulating in the cytoplasm and (4) translocating to the nucleus. (5) Once in the nucleus, β-catenin binds to the T cell factor/lymphoid enhancer factor (Tcf/Lef) family of DNA-binding factors to form a transcriptional complex that binds target promoter sequences via a specific DNA-binding domain in TCFs, mediating Wnt target gene expression. APC, adenomatous polyposis coli.

In the absence of Wnt stimulation, β-catenin is tagged for degradation by a cytoplasmic 'β-catenin destruction complex'. This complex is assembled by the scaffolding protein Axin and comprises glycogen synthase kinase (GSK)3β, casein kinase 1 and the adenomatous polyposis coli (APC) protein and others, which function by capturing 'surplus' β-catenin that is not involved in cell adhesion [122,132]. Once β-catenin is bound by Axin and APC, which act by efficiently positioning β-catenin and the kinases together, phosphorylation of β-catenin occurs by a dual kinase mechanism, whereby phosphorylation by casein kinase I triggers further phosphorylation by GSK3β [141]. This tagging results in β-catenin being recognized by β-Trcp, a ubiquitin ligase, causing β-catenin ubiquitination and subsequent proteasomal degradation [142]. In this manner, despite being continuously synthesized in the cell, the levels of cytosolic β-catenin are kept low, thus preventing it from accumulating in the nucleus. When Wnts bind to Fz transmembrane receptors and low-density lipoprotein receptor-related protein co-receptors (Lrp5 or Lrp6; Figure 3(1)), the scaffolding protein dishevelled (Dvl) joins this receptor complex, causing sequestration of Axin and, thereby, the dissolution of the β-catenin destruction complex [143-147] (Figure 3(2)). Consequently, β-catenin is not marked for degradation and is stabilized as a hypophosphorylated form, accumulating in the cytoplasm (Figure 3(3)), with preferential nuclear localization (Figure 3(4)). Once in the nucleus, β-catenin binds to the T cell factor/lymphoid enhancer factor (TCF/LEF) family of DNA-binding factors, which bind to target promoter sequences via a specific DNA-binding domain in TCFs to form a transcriptional complex that mediates Wnt target gene expression [122,123] (Figure 3(5)). Furthermore, β-catenin is essential as a docking platform for various transcriptional co-activators and chromatin remodeling complexes to stimulate transcription [107,148].

### Wnt signaling crosstalk with key signaling pathways

β-catenin/TCF transcriptional activity can be turned on by signaling molecules other than Wnt proteins (reviewed in [149]). For example, insulin and insulin-like growth factor-1 promote β-catenin nuclear translocation and its binding to TCF on Wnt target gene promoters [150]. β-catenin also interacts with forkhead box O (FOXO) transcription factors to mediate protection against oxidative stress. In this case, FOXO proteins (which are active in stress signaling) will compete with TCF factors (which are active in development and proliferation) for the limited pool of free β-catenin [151-153]. Noteworthy, insulin and growth factors will have a role in this Foxo/TCF competition by antagonizing the function of FOXOs via a phosphorylation-mediated nuclear exclusion process [154]. Finally, cross-regulation of canonical Wnt signaling with that of nuclear receptors) has been observed, including a mutual regulation between Nurr1 and canonical Wnt signaling (for a review, see [155,156]).

## Wnt signaling in CNS development

**Figure 4:**
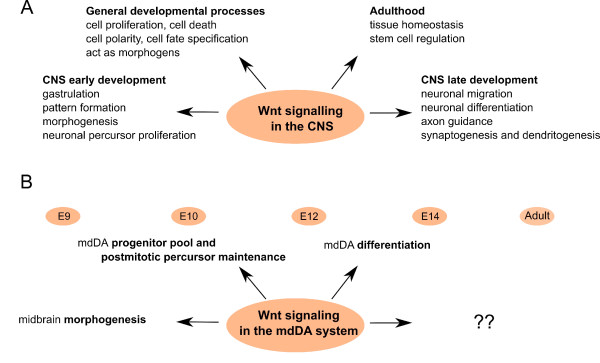
**Wnt signaling during the central nervous system and mesodiencephalic dopaminergic neuron development**. **(A) **Wnt signaling is critical in embryonic development, controlling diverse processes, such as cell proliferation and cell polarity. It is involved during early central nervous system (CNS) development in gastrulation, early pattern formation, morphogenesis and precursor proliferation, in late CNS development in processes such as neuronal differentiation and migration, and in adult organisms, where it plays a central role in the maintenance of tissue homeostasis and stem cell regulation. Wnt signaling controls diverse processes, such as cell proliferation, cell polarity, cell death and cell fate specification Wnts can also function as morphogens in both short- and long-range signaling, modulating target cells in a dose- and distance-dependent manner. **(B) **Wnt signaling is involved in mesodiencephalic dopaminergic (mdDA) neuron development from early on, where it is involved in morphogenesis, and later on as well in mdDA differentiation.

Recent studies have provided us with a more complete picture of the dynamic expression patterns of Wnts, their receptors and co-factors during development and adulthood (for a review, see [157,158]). The expression of Wnt signaling components during development of the CNS has also been described. To begin with, many Fz receptors are expressed in the mouse brain [139,159], including Ryk, the lipoprotein receptor-related protein co-receptor [160] and the receptor tyrosine kinase-like orphan receptor (Ror) family [161]. Secondly, various Wnts are expressed in the developing CNS and peripheral nervous system, in overlapping and complementary patterns [162]. More and more evidence has surfaced corroborating the key role of Wnt signaling in the developing neural tube and brain [117]. Indeed, Wnt signaling seems to take part in most of the processes needed to generate a fully functional neuron (Figure 4A) from a neuronal stem cell, participating in early events, such as neuronal induction [163], anterior-posterior patterning and morphogenesis [164-167] and neuronal precursor proliferation [168-174], as well as in later processes, such as neuronal differentiation in the spinal cord [175], neuronal stem cells [176,177] and cortical neuronal precursors [178], neuronal cortical migration in mice [179], axon guidance in *Drosophila *[180] and in mice [181-183], synaptogenesis [184-186], synaptic differentiation [187,188], dendritogenesis [189,190] and neurogenesis in the telecephalon and hindbrain of adult mice [191-193].

## Wnt signaling in mdDA neurons

Wnt signaling has a known and pivotal impact on mdDA neuron development (Figure 4B). To begin with, midbrain morphogenesis is regulated by Wnt signaling. *Wnt1 *mutant mice present an abnormal posterior midbrain, isthmus and rostral hindbrain, unveiling the essential role of Wnt signaling in MHB formation [194,195]. Other studies support the central role of the canonical pathway in the patterning of the MHB region, where the direct inactivation of β-catenin in a specific manner in the MHB mimics the *Wnt1 *mutant phenotype [170,196]. Furthermore, mutant mice for the Wnt receptor Lrp6 also phenocopy some of the *Wnt1 *mutant defects [160,197] and *Fzd3 *and *Fzd6 *double mutants show a severe impairment of midbrain morphogenesis [198]. In addition, Wnt1 directly regulates the expression of Otx2, a factor involved in midbrain morphogenesis, in a Wnt1-Lmx1a autoregulatory loop during embryonic development [199].

The proliferation and differentiation of midbrain dopaminergic neurons during ventral midbrain neurogenesis depend on Wnt signaling (reviewed in [31,200]). Already during early midbrain development, several members of the Wnt family are expressed and seem to be tightly regulated in a spatiotemporal way [158,159,162,201,202]. In particular, β-catenin transcriptional activity has been observed to take place in the developing mouse midbrain before the birth of Th-positive neurons (at E10.5), with stronger intensity in the Nurr1 expression domain [201]. It seems that activation of the Wnt/β-catenin pathway contributes to increased DA neurogenesis during development: β-catenin promotes midbrain dopaminergic neurogenesis *in vivo *[203] and the stabilization of β-catenin in ventral mesencephalic precursors, by GSK3β inhibition, leads to an increase in DA differentiation [204,205]. In these two recent studies, a targeted deletion of β-catenin in Th-IRES-Cre;β-Ctn^fl/fl ^mutants resulted in reduced mdDA neurogenesis [205]. More recently, the same group reported that the constitutive activation of Wnt/β-catenin signaling in the ventral midbrain of the Th-IRES-Cre;β-Ctn^Ex3/+ ^mutants revealed a significant increase in the number of dopaminergic neurons as well as an increase in the number of committed progenitors, in line with their previous work [206]. On the other hand, activation of β-catenin in Shh-Cre; β-Ctn^Ex3/+ ^mutants led to the expansion of dopaminergic progenitors by reducing their exit from the cell cycle, with a concomitant reduction in the number of dopaminergic neurons more intensely in the SNc [206]. This suggests an opposing role for Wnt/β-catenin signaling in early and late mdDA development. Another study showed that Wnt1 activation enhanced the differentiation of mouse embryonic stem cells to mdDA neurons [199]. Wnt1 was also found to be required for the terminal differentiation of midbrain dopaminergic neurons at later stages of embryogenesis [207]. In addition, Wnt2 was recently identified as a novel regulator of dopaminergic progenitors, necessary in their proliferation; *Wnt2-*null mice therefore have decreased numbers of dopaminergic neurons [208].

In the next two sections we focus on what is known about Wnt signaling in connection to two decisive transcription factors involved in the development of the mdDA neurons, Nurr1 and En1.

### Wnt signaling and Nurr1

**Figure 5:**
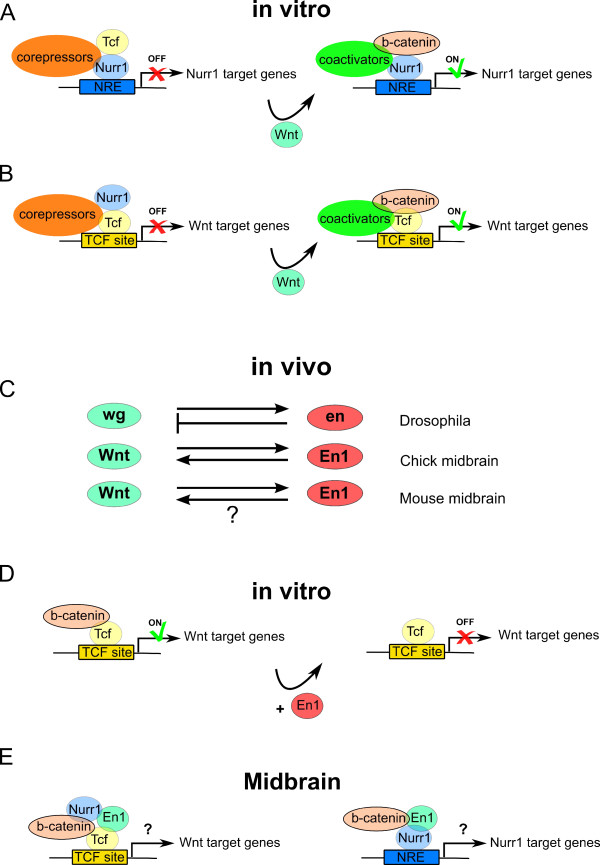
**Interplay between Wnt, Nurr1 and En1 signaling *in vitro *and *in vivo***. **(A) **Model adapted from Kitagawa *et al. *[156]: Wnt signaling via β-catenin enhances the transcriptional activity of Nurr1 in cells at Nurr1 responsive elements (NREs). In the absence of β-catenin, Nurr1 associates with T cell factor/lymphoid enhancer factor (TCF/LEF) in co-repressor complexes on NREs. After activation of Wnt signaling, β-catenin interacts with Nurr1 on NREs, competing with TCF/LEF for Nurr1 binding, resulting in the disruption of the co-repressors from the Nurr1 complex and the concomitant recruitment of coactivators. **(B) **Model adapted from Kitagawa *et al. *[156]: on the other hand, Nurr1 was observed to slightly modulate, in a negative way, the canonical Wnt signaling through association with the TCF/LEF region. After Wnt stimulation, β-catenin competed with Nurr1 for Lef binding on the TCF/LEF promoter site and disrupted Nurr1 binding, promoting Wnt-target gene transcription. **(C) **Several studies in *Drosophila *and chick embryos have described interactions between En1/engrailed (en) and the Wnt/wg signaling pathway whereby engrailed expression is dependent on Wnt/wg signaling and *vice versa*. However, in *Drosophila*, engrailed expressing cells did not have active wg signaling. From mice studies it is known that Wnt signaling regulates En1 expression early in midbrain development. Whether the reverse happens in the mouse midbrain is not known. **(D) **In one cell culture study [216], it was observed that En1 can function as a negative regulator of β-catenin transcriptional activity in a post-translational manner (that is, by affecting β-catenin protein levels only). **(E) **Three questions remain currently unsolved: first, whether En1 cooperates with Nurr1 during mdDA development; second, whether Nurr1, En1 and canonical Wnt signaling cooperate in later stages of mdDA neuron development, such as in mdDA neuron specification; and third, whether Nurr1 and/or En1 regulate canonical Wnt signaling during mdDA neuron development.

It has been shown so far that activation of the Wnt/β-catenin pathway contributes to increased mdDA neurogenesis during development, that is, that it regulates the proliferation and differentiation of ventral mesodiencephalic Nurr1 precursors *in vivo *[203]. Taking into account the data as described, Kitagawa et al. [156] tested the possibility of Wnt signaling regulating Nurr1 activity, and found a convergence between Nurr1 transcriptional regulation and Wnt signaling in cell culture. In short, Wnt signaling via β-catenin enhanced the transcriptional activity of Nurr1 in cells, at Nurr1 responsive elements (NREs), leading to *TH *promoter activation (Figure 5A). In the absence of β-catenin, Nurr1 is associated with Lef1 in co-repressor complexes on NREs. After activation of Wnt signaling, β-catenin interacts with Nurr1 on NREs, competing with Lef1 for Nurr1 binding, resulting in the disruption of co-repressors from the Nurr1 complex and the concomitant recruitment of coactivators, such as CBP (Creb binding protein) [156] (Figure 5A). β-catenin functions, so it seems, as a transcriptional cofactor for Nurr1. Small interfering RNAs targeting Nurr1 abolished CBP and β-catenin association with the NRE in the *TH *promoter [156]. On the other hand, Nurr1 was found to slightly modulate, in a negative way, the canonical Wnt signaling by being able to associate with the TCF/LEF region (Figure 5B). After Wnt stimulation, β-catenin would compete with Nurr1 for Lef1 binding on a TCF/LEF promoter site, such as the cyclin D1 promoter, and disrupt Nurr1 binding, promoting Wnt-target gene transcription [156]. A model to describe this mechanism was proposed and is shown in Figure 5A,B. Whether this model is valid for mdDA neuron differentiation and maintenance *in vivo *remains to be investigated. The question arises: do Nurr1 and β-catenin interact *in vivo *synergistically to drive Th expression? Previous studies strongly suggest that this is the case [204-206]. Importantly, besides the study from Kitagawa *et al*., synergistic interactions between β-catenin and several nuclear receptors have already been described [155]. Quite likely, β-catenin is involved in mdDA neurogeneis, cooperating with the Nurr1 transcription complex.

### Wnt signaling and En1

In *Drosophila *and chick embryos, interactions between engrailed (en) and Wnt/wg signaling pathways have been described whereby engrailed expression is dependent on Wnt/wg signaling and *vice versa *[209-211] (Figure 5C). However, in *Drosophila*, engrailed expressing cells did not have active wg signaling [209]. A modulation of Fz receptor expression by engrailed was shown in *Drosophila *wherein the expression of Fz is lower in engrailed-positive domains and, in the *engrailed *null mutant, the usual striped expression of Fz is disturbed, spreading everywhere in a non-segmental pattern [212]. Later, by means of chromatin immunoprecipitation (ChIP) assays, engrailed was established to be a direct repressor of Fz2 expression *in vivo *[213]. In mice, Wnt1 expression was found to overlap with *En1 *gene expression in the midbrain at 8.5 days post-coitus [65,214,215], but while Wilkinson *et al. *[214] found Wnt1 expression in the midbrain after 12 days post-coitus, Davis and Joyner [65] did not observe overlapping expression domains between En1 and Wnt1 within the midbrain after this time point. As an explanation for this discrepancy they advance the fact that Wnt1 expression is punctuated, making it hard to get brain slices containing visible expression. So, a more detailed analysis of the Wnt/β-catenin signaling in the mdDA system is needed. As mentioned above, inactivation of the *Wnt1 *gene leads to the deletion of the midbrain-hindbrain area with concomitant loss of En1 (its expression in the MHB region is initiated normally but is subsequently lost) [194,195,215]. Furthermore, the expression of En1 under the Wnt1 promoter rescues most of the Wnt1 phenotype, suggesting that En1 is a downstream target of Wnt1 [91] (Figure 5C). In conclusion, both Wnt1 and En1 cooperate in the patterning of the MHB region during early development.

In cell culture studies, it was observed that En1 can function as a negative regulator of β-catenin transcriptional activity in a Gro/TLE-independent manner (TLE: transducin-like enhancer of split 1) [216] (Figure 5D). Silencing En1 expression using small interfering RNA stimulated β-catenin transcriptional activity, measured by luciferase reporter assays. By Northern analysis and cycloheximide assays, Bachar-Dahan *et al. *[216] observed that En1 affects the level of a constitutively active form of β-catenin at a post-translational level only. They suggest that En1 acts by destabilizing β-catenin via a proteasomal degradation pathway that is GSK3β-independent [216].

As we mentioned above, there might be a link between En1 depletion and the onset of neurological disorders such as PD. A direct interaction between the PD-associated protein parkin and β-catenin has recently been observed [217]. In this study, increased levels of β-catenin activity were found in *parkin *mutant mice. This increase in Wnt-β-catenin signaling led to an increase in dopaminergic neuron proliferation and death [217], which is in contrast to the positive role Wnt-β-catenin signaling plays during mdDA neuron development. This might be due to the different needs in Wnt signaling activity in morbid adult dopaminergic midbrain tissue when compared to healthy one [158]. It is currently unknown whether En1 and canonical Wnt signaling cooperate in later stages of mdDA neuron development, such as in mdDA neuron specification and maintenance (Figure 5E).

### Conclusions and future perspectives

The vertebrate mdDA system has been intensively studied in the past decades and an enormous wealth of information on the molecular cues controlling its development has been gathered. Our future challenge is to unravel in depth the gene cascades linking early induction to the differentiation and maintenance of mdDA neurons, eventually obtaining a complete picture of mdDA development (including the developmental origin and the molecular coding characterizing various mdDA subsets). Once this is accomplished, effective clinical treatments for mdDA-associated neurological disorders, such as PD, can be generated. As described in this review, current evidence strengthens the central roles that En1 and Wnt signaling might play in the advancement of these therapies, especially for PD. However, a detailed molecular characterization of the *En1 *mutant is lacking. Furthermore, the precise function of En1 in some mdDA developmental processes is also not known, and questions such as whether En1 is essential in the differentiation of the mdDA system and is part of key transcriptional complexes mediating such processes (such as the Nurr1 complex) need to be investigated.

Concerning Wnt signaling, detailed knowledge about which developmental processes it regulates within a particular dopaminergic neuron, as well as which key players are involved, is still incipient. Furthermore, a more detailed characterization of Wnt/β-catenin activity during ventral midbrain development is essential. Wnt signaling overlaps with that of En1 in time and space during CNS development and these two pathways interact functionally at least at one time point during embryonic mdDA development. It is now known that canonical Wnt signaling and En1 cooperate in the genesis of a competent mdDA field during early development. However, whether Wnt signaling and En1 might cooperate in later midbrain developmental stages, such as in the differentiation of mdDA neurons, is still not known. Future research will have to focus on disclosing the *En1 *mutant phenotype and its relevance and eventual interplay with Wnt signaling during mdDA differentiation. Recent improvements in techniques, such as transcript expression profiling, ChIP-seq, proteomics, and mdDA neuronal cell isolation and culture, will certainly help unveil the molecular repertoire necessary to generate a mdDA neuron.

## Abbreviations

A/P: anterior-posterior; ChIP: chromatin immunoprecipitation; CNS: central nervous system; DA: dopamine; E: embryonic day; En: Engrailed; Fgf: fibroblast growth factor; FOXO: forkhead box O; Fz: Frizzled; GSK: glycogen synthase kinase; LEF: lymphoid enhancer factor; mdDA: mesodiencephalic dopaminergic; MHB: midbrain-hindbrain boundary; NRE: Nurr1 responsive element; PD: Parkinson's disease; Shh: sonic hedgehog; SNc: substantia nigra pars compacta; TCF: T cell factor; Th: tyrosine hydroxylase; VTA: ventral tegmental area.

## Competing interests

The authors declare that they have no competing interests.

## Authors' contributions

MTMAS and MPS carried out the writing of the manuscript. Both authors have read and approved the final manuscript.
